# From Our Correspondents

**Published:** 1989-02

**Authors:** 


### From our correspondents

### The White Paper—Working for Patients

A general practitioner's freedom to provide care for his or her patients is under threat following the publication of the government's new White Paper 'Working for Patients'. To date, general practitioners have exercised their freedom in various ways and at different costs to the NHS. Some refer more frequently to specialists than do others, the reasons given for this vary from wanting to give patients the benefit of an expert opinion to prefering a specialist to take over a task that most generalists believe should be performed by themselves. On the other hand some doctors naturally refer infrequently; do they take too much on or are they ignorant of the possibilities of specialist care? Prescribing also has few restrictions; if a specialist or general practitioner believes medication is appropriate this is provided regadless of cost. The same applies to diagnostic tests performed by hospital laboratories or departments of radiology.

Each general practitioner has a varying understanding of the costs he or she is imposing on the NHS. Regularly a GP receives information about the cost of the previous quarters' prescribing compared to that of the national and FPC averages. On the other hand, he receives no information about the frequency of hospital referrals nor his frequency of asking for diagnostic tests and has no idea how his rates compare with his neighbours. Each general practitioner holds unique views on what he should or should not provide for his patients; some believe that all patients should have the right to every service the NHS provides, others that they should be encouraging self-sufficiency amongst their patients. Some are interventionist, others cautious.

At present the NHS copes with all of these different approaches to patient care with varying success. The White Paper however proposes a major change. General practitioners will have to add a new dimension to each clinical decision they make. What is its cost and can it be afforded?

Those of us who have worked in other countries have often experienced the dilemma of whether a patient can afford a procedure and whether we should persuade impecunious or uninsured patients to undergo certain expensive procedures or treatments. That this type of decision has to be shared with a patient is bad enough, but now we are asked to decide whether our personal budgets will enable the patient to have a procedure performed. Learning to share that dilemma with his patient is likely to prove difficult, and many doctors may simply not mention the possibility of treatment or diagnostic test to the patient, preferring to choose the cheaper option. Doctors are often attracted to general practice because it is like a little business. Over time you get to know your clientele, you employ your own staff, you arrange your own premises and have practice accounts. You have the freedom to add to your income by working in hospitals, local industry or teaching the next generation of doctors. It seems that the government is banking on this commercial aspect of general practice to persuade doctors to extend their role of gatekeeper to the NHS to that of manager of the NHS. The government is able to make this change very attractive to general practitioners but whether we should accept the bribe is questionable. I believe the government is right to confront the profession with this dilemma at this time. We need to know that our actions impose costs on the NHS and we need to know what these costs are. Having more information should enable us to monitor our activities more closely and to ensure that quality of care is rising. Many of us will certainly require help in coping with these changes. The imposition of sanctions on those doctors who consistently cost the NHS more than their colleagues may be necessary as a last resort. However, I believe that if general practitioners accept budgets as described in the White Paper, we shall change our relationship with our patients dramatically and for the worse.

If we refuse to accept budgetting the government will inevitably claim we are conservative and unwilling to change, but that is not the case. We encourage the government to provide efficient monitoring systems for prescribing and referrals, we have been doing so for years, to little effect. We encourage the development of audit. We have been doing so for years. We accept the need for help, and possibly, sanctions on persistent 'overspenders', but we fail to see the benefit of developing the budgetting infra-structure in general practice as envisaged in the White Paper.

Michael Whitfield

### The New G.P. Contract—A Minority View

The new contract for G.Ps. arrived on the doorstep closely behind the White Paper proposals. It came as a great cultural shock to the vast majority of family doctors. After the teachers and lawyers, the last professional monopoly to be challenged was the medical profession.

Given the time scale, April 1st 1990, many of the proposals were going to be difficult to implement, especially the target figures for immunisations and cervical smears. Concurrent reduction in Basic Practice Allowance and the disappearance of several other allowances could well leave some practices, including the writer's, financially worse off.

However the philosophy behind the new contract was unarguable. It certainly contained many of the aims and aspirations of the Royal College of G.Ps. This was the first time in 40 years of the N.H.S. that any government had attempted to make family practitioner services more consumer orientated. Who can doubt that from the patient's point of view there are many advantages?

It will be easier to change doctors. Practices will have to produce leaflets giving the services offered and other details. There will be more money for doctors who do their own night work and for doctors who carry out their own minor surgery, thus saving patients time by avoiding a hospital referral. Family Practitioner Committees will be given greater powers to oversee and monitor acceptable standards of premises, equipment and staff training. Prescribing costs will be watched closely (currently running at twice the gross income per G.P.) and hopefully will eliminate unneccessary and profligate prescribing which will be very much in the patient's interests. Compulsory retirement of G.Ps. will be implemented at 70 years and soon brought down to 65 years. Routine surveillance of young and old will be part of our contract and medical examinations will be offered to all new patients on a G.P's. list. Finally it is proposed that all G.Ps. live with reasonable distance of their practices, that they will take annual postgraduate refresher courses and produce annual practice reports for the F.P.C. This must be welcome news for the patients though not necessarily for the doctor.

I have not touched on several other aspects of the new contract as space will not allow me. Budget holding for G.Ps. is an innovation which will not be for the fainthearted but strictly for the entrepreneurs! The Health Department has made it quite clear it is voluntary. Only large and very well organised practices could be considered candidates, most of us will be preoccupied with the new proposals for the next few years.

The taxpayer, the patient and the government have a perfect right to expect value for money, uniform standards of care and accountability. I have no doubt that this will be achievable in the next 2–3 years.

At the time of writing, after much sabre rattling by the profession, a settlement has been reached with the health department on the amended contract by our negotiators. This will be put before the profession at the Annual Conference in June. Already there is more talk of resignation. This is totally unjustified. The amended contract is live-able with and, when implemented, will provide a better overall service to patients and great opportunities for keen, able, well organised and energetic G.Ps.

Jeremy P. Telling,

**Revolutions, Quiet and Noisy**

Revolutions can be quiet or noisy and Cornwall has experienced both. The quiet one has been the gradual disposal of mental health care from large institutions into the community. That it has been quiet is a tribute to the tact and expertise of those who have effected the changes, for example an acting DMO who smuggled through a report on the resettlement of the mentally handicapped at the end of an ineffably boring MEC meeting and did it so skilfully that nobody even read beyond the first page. The integration of these ex-patients into the wider world is going smoothly and sheltered domestic houses are appearing around the country at the rate of one every two months.

In addition, nearly 400 out 900 beds at St. Lawrence's Hospital (the old Bodmin asylum) have been closed; a dawn raid in the Administration acquired the old SWEB building in Redruth to give in-patient facilities in the west of the country whilst psycho-geriatric patients (now known as EPDs and the majority group in any large psychiatric hospital) are being further dispersed into small units closer to their homes. By the mid 1990s the base hospital should be down to 250 beds, mostly for acute assessment and treatment. One is left with a little semantic puzzle, Why is Community such a Good Thing whilst the word Society has been expunged from the Newspeak Dictionary?

The noisy revolution has been in the acute services unit where two major capital developments are now under way. Planning of the new DGH at Truro started only in the 1950's and building has now about passed the half way stage. The new development will reunite obstetrics and gynaecology which are currently seven and a half miles from each other and a custom built accident and emergency department will replace one which would have disgraced the orlop deck of the Victory. This will still leave a DGH spread over six or eight sites, depending on your view of what a DGH should contain, and over a radius of twenty miles. There is still no Out-Patient Department which has been tentatively pencilled in for some time in the next millenium.

As a bonus, and as the result of some Machiavellian manoueverings at region, it has been possible also to make a start on the new theatre suite at the West Cornwall Hospital in Penzance. This will replace the existing theatres which date from the early nineteen thirties and it opens the way for a planned major expansion on the site.

Both these developments have been fought for and are welcomed with rejoicing although one should not forget that there is an element of embarrasement and shame that we cannot move a bit faster.

M. L. Crosfil

**Report on the 8th World Congress of the International Association for the Scientific Study of Mental Deficiency**

The 8th World Congress of the International Association for the Scientific Study of Mental Deficiency took place at Trinity College, Dublin, Eire, from 21st to 25th August 1988, co-sponsored by the World Health Organisation. The theme of the Congress was 'Key Issues in Mental Retardation Research'. There were over 1,400 delegates attending from 45 countries, with many different disciplines being represented. The inspiring surroundings of Trinity College were matched by some equally stimulating scientific papers on topics of current interest in mental handicap.

The issue of prevention was very much to the fore, and the different aspects of this which included:- socioeconomic factors, genetic counselling, prenatal diagnosis and antenatal care including the prevention and treatment of infections both pre and post natally. One interesting issue raised by a representative from the United States was that of infants who are born HIV positive. At present their lifespan is approximately 18 months but if medical advances mean that their survival is prolonged, a large number of them will be mentally handicapped. Other possible new causes of mental handicap were discussed, such as alcohol abuse and the new generation of women with treated phenylketonuria, who are now starting to have children of their own. Current policies of community care for the mentally handicapped will result in greater opportunity for those people with a hereditary disorder such as Fragile X syndrome to pass it on to future generations. Papers from several countries, including the U.S.A. and Australia, warned of the dangers of closing hospitals before adequate services in community have been developed.

Another current topic arousing much interest is that of the medical aspects of care of the mentally handicapped, particularly in association with ageing. So much research is being done currently, particularly on the association between Down's syndrome and Alzheimer-type dementia, that it is hopeful that some answers will be found that will benefit the ageing population as a whole. The increasing longevity of mentally handicapped people has serious implications for future service provision and much work is currently being undertaken to discover more about the medical and psychiatric needs of this group of patients. The Symposium on Ageing in the Mentally Handicapped, sponsored by Frenchay Health Authority and arranged by Dr. J. Jancar in April 1987, made an important contribution to the work in this field.

Four delegates from Stoke Park Hospital presented papers related to this subject. All the papers were well presented and aroused a lot of interest. It was very pleasant to be reminded by delegates from other countries of the good name Stoke Park has internationally in pioneering patterns of care for the mentally handicapped, and in promoting well respected research.

At the Assembly it was announced that Dr. Jancar was elected Honorary Officer of the Council for his past services to the Council and the IASSMD. It was also announced that Dr Jancar with Dr Annalise Dupont, Norway, Professor Zena Stein USA, and Dr Andrews, Australia, were nominated for membership of the Joint Commission on International Aspects of Mental Retardation with the World Health Organisation.

Leila B Cooke.

**Fishing doggo**

Government white papers abound. Most correspondents will no doubt respond. This one feels too bemused by yet further reorganisation. There are times when one wonders if life is as it should be. One of these times is when out entertaining someone else. People are usually quite good at being entertained. Dogs are more fastidious. Most town dogs tire quickly and yet need constant entertainment.

A few weeks ago I found out that pets are not easily mixed in with hobbies. I have recently acquired a splendid Welsh Springer spaniel with a pedigree much better than my own. Yes, thank you, she is in excellent health. For those given to civilised and sedentary weekends in town, perhaps I ought to explain that these dogs are the sort that take you out for three hour walks in muddy fields and across duck ponds, with a quick canter through apparently inpenetrable hedges to finish the proceedings in search of sheep, deer or whatever remaining game that has been too coy in the preceding two and a half hours. If not already wet enough Springers usually then manage to find a canal close enough to home to guarantee that they are scarcely in a fit condition to join wives or daughters delicately enjoying an afternoon cucumber sandwich or two. As you can imagine wives and daughters are firmly of the opinion that such dogs are your responsibility. They—the dogs I mean—thereafter take immense delight in coaxing you to play ball when slumped in a state of ashen-faced collapse in front of the fire downstairs well out of the way.

Anyway, what with one thing and another—largely because the Welsh rugby team were clearly losing badly at the Parc des Princes, I was allowed out at half time to go fishing. You can imagine the sly female rejoinder: 'Do take the dog as well, she'll enjoy the trip'.

The time of year was what is jokingly called Spring Time in salmon fishing circles. No-one in their right mind would think of calling the combination of horizontal sleet, rain, grey-black clouds and light that is reminiscent of the more impressive solar eclipses in the far Pacific spring, but there you are. Out we dutifully go. Waders, sinking line and tube flies.

Sensible folk would balk at this. So did I.

Once on the river side, the scene was visibly far worse than on the rugby field in Paris. A sort of liquid milk chocolate with white horses, chunks of trees, the occasional dead ewe and lashing spray was raging down the main stream. Happily I have a normally quiet side stream on the fishing beat which is normally an easily fordable and placid series of pools with a gentle trickle keeping them topped up. On that afternoon it too was in turmoil. A sort of kindergarten practicing civil war. However we crafty fisherman appreciate the feelings of salmon. They also prefer peace and quiet. It seemed a good place to try the odd cast or two. At the outset it must be said in general that dogs do not mix with fishing tackle. There are few things which I have got my dog to agree upon, but this principle is one. When master is fishing spaniels must find their own pursuits. No, not sheep chasing, but gentler activities like demolishing mole mounds or even a little gentle paddling.

So far so good. Master happy—more or less—and dog digging.

It was at this point that things started to go wrong. Until then I had no idea at all why dogs had tails. However, just as the casting was really beginning to get into a decent rhythm I happened to see at 45 degrees left an animal launching into the spate. It was really like watching a slow-motion action replay film of someone else's life. The odd thing was that this animal was obviously well bred and had a curiously familiar vivid red and white fur. It certainly had no clear idea as to the need for swimming when under water. Masters at this time are clearly men of immediate and decisive action. With the side swerve lacking amongst the Welsh three quarters earlier that afternoon it was the work of a moment to grasp the last part of the dog, which was in any case by now blowing bubbles in the milk chocolate. Frankly I was astonished how solid a structure the tail of a dog is.

Kind relatives later that evening—when they squeezed out the story—asked whether the water wasn't cold. To my surprise, I couldn't really remember. All I do recall is that although the dog was miraculously restored to the bank, master was now fast finding that it was as well that he had gone to the trouble to put on chest waders. I should like to tell that dog repaid the kindness by pulling master out. Far from it. She was more concerned with the forbidden rod, and managed with astonishing dexterity to unseat the reel which quickly followed into the torrent.

It is true though that when wet master eventually managed to regain the bank some yards downstream thanks were expressed. Oddly this took the form of copious licking, muddy paws all over the place, and a firm determination to be an otter. Master by now had learnt the lesson and yanked a tail with rather less concern than previously.

However relationships were soon reeastablished. The only passing problem remained as how to get this sodden and muddy mixture of dog, master and tackle into what at one time used to be an agreeably dry if temperamental car. It is probably best to draw a discrete veil over that interlude.

Message: dogs are best off at Crufts. Cucumber sandwiches also have their appeal.

J. D. Davies

